# Supplementary material for: Inhibiting Effects of Ginger and Rosemary on the Formation of Heterocyclic Amines, Polycyclic Aromatic Hydrocarbons, and Trans Fatty Acids in Fried Pork Balls
Source: Foods. 2022 Nov 23;11(23):3767. doi: 10.3390/foods11233767 (PMC9738988; doi:10.3390/foods11233767)
Supplement: Supplementary file 1 [file foods-11-03767-s001.zip › foods-2011741-supplementary.pdf]

Supplementary

Table S1. The test method verification results

	Linear equation	R^2	LOD (μg/L)	LOQ (μg/L)
Norharman	Y=41749X-839.188	0.999844	0.0065	0.0217
Harman	Y=72078.7X-738.068	0.999648	0.0042	0.0140
AαC	Y=25646.8X-109.537	0.999120	0.0062	0.0206
MeAαC	Y=17985.8X-172.078	0.999125	0.0087	0.0290
IQx	Y=12824.7X-572.394	0.999735	0.0619	0.2060
MeIQ	Y=11136.1X-617.139	0.999563	0.0224	0.0746
MeIQx	Y=5620.43X-914.233	0.999062	0.0632	0.2108
PhIP	Y=27555.7X-168.412	0.999804	0.0072	0.0240
7,8-DiMeIQx	Y=20325.7X-476.537	0.999160	0.0101	0.0336
Trp-P-2	Y=31011.2X-211.842	0.999626	0.0058	0.0194
4,8-DiMeIQx	Y=18502.6X-371.087	0.999247	0.0158	0.0527
BaA	Y=692.85X-150.42	0.9995	0.33	1.08
Chr	Y=378.48+1620.9	0.9994	0.53	1.78
BaF	Y=665.45X+594.11	0.9991	0.62	2.07
BaP	Y=395.84X+389.52	0.9994	1.35	4.51
